# Perceptions of women on seeking sexual healthcare in Beirut: a qualitative study

**DOI:** 10.1080/26410397.2026.2639175

**Published:** 2026-03-04

**Authors:** Diana Sabbagh, Jessie V Ford, Sohayla El-Fakahany, Faysal El-Kakd

**Affiliations:** a Research Assistant, Faculty of Health Sciences, American University in Beirut, Beirut, Lebanon; b Assistant Professor, Department of Sociomedical Sciences, Columbia University, New York, NY, USA.; c Social Research Consultant, American University of Beirut, Beirut, Lebanon; d Associate Professor, Clinical Obstetrics and Gynecology and Public Health and Director of Women Integrated Sexual Health (WISH) Program, American University in Beirut, Beirut, Lebanon

**Keywords:** sexual and reproductive health and rights, women’s perceptions, access to sexual healthcare, sexual health services

## Abstract

Sexual health is a vital component of overall well-being, shaped by biological, emotional, and sociocultural influences. In Lebanon, the limited provision of sexual healthcare for women remains a neglected concern that perpetuates inequities in access and outcomes. Understanding women’s perceptions of sexual healthcare, alongside provider perspectives, is essential to designing effective interventions and strengthening health systems. This study examines Lebanese women’s experiences and expectations regarding sexual healthcare seeking. A qualitative study was conducted in Beirut (2022–2023) using in-depth interviews with 19 participants: 6 women who had sought sexual health services, 8 who had not, and 5 healthcare providers. Data were analysed thematically using an inductive approach, with codes generated and compared within and across interviews to build broader thematic categories. Three overarching themes emerged. (1) *Barriers to Care*: participants described stigma, cultural restrictions, fear of judgment, poor communication with providers, and limited service options as central obstacles. (2) *Facilitators of Care*: awareness of available services, sexual activity, supportive relationships, medical needs, and positive physician–patient interactions enabled access. (3) *Recommendations*: participants highlighted expanding service availability, strengthening awareness campaigns, and training providers to offer sensitive, non-judgmental care. Despite cultural and systemic barriers, Lebanese women show willingness to seek sexual healthcare when it is accessible, acceptable, and supportive. Improving sexual healthcare requires widening access, enhancing education, and building provider capacity to deliver woman-centred care. Findings underscore the need for policy, practice, and research initiatives that foster open dialogue, equitable access, and integrated services tailored to women’s needs.

## Plain language summary

Sexual and reproductive health is a vital part of women’s overall health and well-being, yet it is often overlooked in Lebanon. This study explores the experiences of women in Beirut when accessing sexual healthcare, aiming to understand the factors that make it easier or harder for them to get care. We interviewed women who use sexual health services, those who do not, and the physicians who provide these services. Our findings show that women’s decisions to seek care are shaped by multiple factors. Social stigma, fear of judgment, poor communication from providers, and limited service options act as barriers. Knowledge about sexual health, being sexually active, having a medical condition, and respectful communication with providers motivate women to seek care. Women recommended improving awareness of available services, ensuring privacy, and making care affordable. Listening to women’s voices can help design sexual health services that truly meet their needs.

## Résumé

La santé sexuelle, élément essentiel du bien-être global, est influencée par des facteurs biologiques, émotionnels et socioculturels. Au Liban, l’offre limitée de soins de santé sexuelle pour les femmes demeure un problème négligé qui perpétue les inégalités en matière d’accès et de conditions de santé. Il est indispensable de comprendre la perception qu’ont les femmes des soins de santé sexuelle, ainsi que le point de vue des professionnels de santé, pour concevoir des interventions efficaces et renforcer les systèmes de santé. Cette étude examine les expériences des Libanaises et leurs attentes concernant la demande de soins de santé sexuelle. Une étude qualitative a été réalisée à Beyrouth (2022-2023) au moyen d’entretiens approfondis avec 19 participants: six femmes qui avaient eu recours à des services de santé sexuelle, huit qui ne l’avaient pas fait et cinq professionnels de santé. Les données ont été analysées thématiquement à l’aide d’une approche inductive, avec la création et la comparaison de codes au sein des entretiens et entre ceux-ci pour dégager des catégories thématiques plus larges. Trois thèmes généraux sont apparus: (1) obstacles aux soins: les participants ont décrit la stigmatisation, les restrictions culturelles, la peur du jugement, la mauvaise communication avec le personnel soignant et le nombre limité de services proposés comme principaux obstacles; (2) facteurs facilitant l’accès aux soins: la connaissance des services disponibles, l’activité sexuelle, les relations de soutien, les besoins médicaux et des interactions positives entre le médecin et la patiente ont facilité l’accès aux soins; (3) recommandations: les participants ont souligné combien il est important d’élargir l’offre de services, de renforcer les campagnes de sensibilisation et de former les prestataires à assurer des soins respectueux et sans jugement. En dépit d’obstacles culturels et systémiques, les Libanaises se montrent disposées à recourir aux soins de santé sexuelle quand ils sont accessibles, acceptables et bienveillants. Pour améliorer les soins de santé sexuelle, il faut élargir l’accès, améliorer l’éducation et renforcer les compétences des prestataires afin qu’ils puissent dispenser des soins centrés sur les femmes. Les résultats de l’étude soulignent la nécessité d’initiatives politiques, pratiques et de recherche qui favorisent un dialogue ouvert, un accès équitable et des services intégrés adaptés aux besoins des femmes.

## Resumen

La salud sexual es un componente vital del bienestar general, en el que influyen factores biológicos, emocionales y socioculturales. En el Líbano, la prestación limitada de servicios de salud sexual para las mujeres continúa siendo un asunto olvidado que perpetúa las inequidades en el acceso y en los resultados. Para diseñar intervenciones eficaces y reforzar los sistemas de salud, es esencial entender las percepciones de las mujeres de los servicios de salud sexual y las perspectivas de los prestadores de servicios. Este estudio examina las experiencias y expectativas de las mujeres libanesas con relación a su búsqueda de servicios de salud sexual. Se realizó un estudio cualitativo en Beirut (2022–2023) utilizando entrevistas a profundidad con 19 participantes: seis mujeres que habían buscado servicios de salud sexual, ocho que no los buscaron y cinco prestadores de servicios. Se analizaron los datos temáticamente utilizando el enfoque inductivo, con códigos generados y comparados en y entre entrevistas para crear categorías temáticas más amplias. Surgieron tres temas generales: (1) *Barreras a los servicios*: las participantes describieron el estigma, las restricciones culturales, el miedo a ser juzgadas, mala comunicación con prestadores de servicios y opciones limitadas de servicios como obstáculos centrales. (2) *Facilitadores de los servicios*: conocimiento de los servicios disponibles, actividad sexual y relaciones de apoyo, necesidades médicas, e interacciones médico-paciente positivas facilitaron el acceso. (3) *Recomendaciones*: las personas participantes destacaron ampliar la disponibilidad de los servicios, fortalecer las campañas de sensibilización y capacitar a prestadores de servicios para brindar atención sensible y sin prejuicios. A pesar de las barreras culturales y sistémicas, las mujeres libanesas muestran buena disposición a buscar servicios de salud sexual cuando estos son accesibles, aceptables y solidarios. Para mejorar los servicios de salud sexual es necesario ampliar el acceso, mejorar la educación y desarrollar la capacidad de los prestadores de servicios para brindar atención centrada en las mujeres. Los hallazgos subrayan la necesidad de políticas, prácticas e iniciativas de investigación que fomenten un diálogo abierto, acceso equitativo y servicios integrados adaptados a las necesidades de las mujeres.

## Introduction

Sexual health is a core element of overall health that includes physiological, emotional, and sociocultural factors.^1^ Achieving improved sexual health requires adequate knowledge about sexuality and associated risk factors, as well as access to care in a supportive context.^2^ Sexual healthcare (SHC) is part of the broader field of sexual and reproductive health (SRH), which includes services such as family planning, prevention and treatment of sexually transmitted infections, and support for sexual well-being. SHC incorporates a broad range of preventive and therapeutic services such as sexual health counselling, screening, prevention, and cervical cancer screening with pap smears and HPV vaccinations. In addition, SHC encompasses the assessment and management of sexual dysfunction as well as sexual health needs and well-being during key periods such as before and after birth.

Although sexual health has received more attention in recent years, it is still frequently overlooked in lieu of other priorities.^3^ Sexual and reproductive healthcare needs are particularly underserved in the Extended Middle East and North Africa (MENA) region in countries like Lebanon, Jordan, Egypt, Morocco, Tunisia, and Gulf states like Saudi Arabia and the United Arab Emirates, despite the importance of the availability of these services.^4,5^ For instance, in countries like Egypt and Lebanon, SRH services are mostly implemented as a part of private gynaecology and obstetrics clinics for married women.^6^ Although several Arab countries have undergone improvements in healthcare, which include the integration of family planning into primary care,^7^ the development of national adolescent SRH strategies (in Jordan),^8^ comprehensive HIV programmes (in Oman),^5^ and the integration of sexually transmitted infections (STI) screening programmes (in the UAE),^6^ we have a long way to go before SRH services are seamlessly incorporated, particularly in settings dealing with or surrounded by economic and humanitarian crisis.^9^

Despite achievements in terms of maternal health, contraception and HIV, a holistic view of SRH that incorporates sexuality, abortion, infertility, other STIs, psychosexual counselling, sexual dysfunction, and gender-based violence is not widespread in Lebanon specifically and in the Global South more generally.^10,11^ While there is ongoing advocacy for women’s sexual rights and gender equality, entrenched social norms, patriarchal structures, and persistent stigma continue to impede progress towards advancing women’s sexual health in the MENA region.^12^ Thus, sexual difficulties encountered by women are often overlooked and minimised in healthcare settings, in part, because women are unaware that this is a legitimate medical issue and because they feel ashamed to talk about it.^3,13^ A study on women’s sexuality in the Middle East revealed that 59% of participants report experiencing disorders related to low sexual desire and arousal that have not been treated because they did not access care due to stigma, shame, and discomfort around sexual health.^11^

While Lebanon is often seen as more progressive around SRH than other countries in the MENA region, studies reveal that knowledge of sexual health and STI/HIV remains limited, constrained by conservative norms that prioritise female virginity and strong societal taboos around sexuality.^12^ In this context, sexuality is most acceptable in marriage, which drives under-reporting of health issues and avoidance of SRH services for unmarried individuals.

Data in Lebanon show that 31% of women experienced intimate partner violence and that these rates were even higher during COVID-19 and periods of political instability, which exacerbated gender inequalities.^14^ For instance, one study found that reported cases of domestic violence have doubled to 1468 cases in 2020 compared to 747 during the previous year.^15^ In 2022, almost 1400 new cases of gender-based violence were recorded in Lebanon.^16^ Other challenges in this context range from internal conflict to severe financial and economic instability, resurgences of the pandemic, and the Beirut Port explosion, which all disproportionately affected women.^17^ Increased household stress, poverty, and declining living conditions increased the risk of intimate partner violence. Also, lockdowns trapped some women with abusers, causing more physical assault, psychological abuse, and sexual violence.^17^

Alongside heightened risks of violence, women in Lebanon also face enduring challenges related to early marriage, HIV, and limited sexual health services. Early marriages are still prevalent; 6% of girls and women marry before the age of 17. The estimated number of adults living with HIV in Lebanon reached 2700 in 2020, with 200 new incident cases,^17^ HIV testing rates are low among women due to stigma and normative ideas about “at-risk” populations; as a result, many women do not access services to avoid poor treatment or ridicule from providers.^14^ Neglecting women’s sexual health in Lebanon hinders access to education and services and increases women’s risk of unplanned pregnancies, unsafe abortion, STIs, violence, sexual disorders, and maternal or neonatal morbidity and mortality.^18^ A recent study documenting the prevalence of various STIs among 351 women attending gynaecology and obstetrics clinics in Lebanon revealed a high prevalence of HPV reaching 15.7%, 6.8% for urea plasma, 13.7% for candida (yeast), and 20.5% for mycoplasma, and a prevalence for gonorrhea, HSV-2, and chlamydia reaching 0.3%, 0.6%, and 2.8% respectively.^19^ Another study of 2,083 sexually active unmarried women found a 15% prevalence of STIs, with 11% of participants reporting an unwanted pregnancy.^20^ Given that abortion is illegal in Lebanon, no accurate statistics are available. In 2023, 24 maternal deaths were reported, with a maternal mortality ratio of 23.78 per 100,000 live births, while neonatal mortality reached 11.2 per 1000 live births during the same year.^21^

To date, studies in Lebanon consistently indicate a need for more sexual health education and training for providers and students^22–24^ and a need for more comprehensive sexuality education.^6,14^ Previous research conducted with Lebanese women has examined: (1) sexual and reproductive health knowledge among unmarried Lebanese women,^4^ (2) interpretations of sexual difficulties and narratives of self-blame related to being in middle age,^3,25^ (3) prevalence of STIs, sexual practices, and substance use among sexually active unmarried women in Lebanon,^26^ and (4) barriers to HIV testing in Lebanon.^18^ Findings from these studies show difficulty in expressing sexual needs, marital conflicts, hidden unsatisfactory sexual experiences due to social expectations, and low SRH-related knowledge. Findings also show that women’s sexual identity is shaped by their husbands’ needs and a prioritisation of the family over self. Sex is considered a taboo topic in this context, limiting open discussion and awareness, complicating policies such as insurance coverage for HIV testing. The majority of studies in Lebanon to date have been epidemiological in nature and lack an in-depth focus on factors influencing or hindering sexual healthcare practice. Building upon past work in hopes of contributing to research on SRHR in the Global South, this study explores Lebanese women’s perceptions around sexual health care seeking and their expectations for these services. Since sexuality is a sensitive, multifaceted construct, it is important to examine it in the context of women’s lives, experiences, values, and beliefs. A greater understanding of the facilitators, barriers, and reasons for accessing care will guide interventions for programme enhancement and expand our understanding of pressing issues related to this context.

## Methods

### Study design

This exploratory study aimed to capture women’s perceptions regarding SHC-seeking and expectations from these services. We also examine healthcare providers’ perspectives to contextualise and better understand how service delivery impacts women’s experiences. We employed a qualitative study design using in-depth interviews to explore women’s emic understandings and lived experiences regarding this sensitive topic and to triangulate these findings with accounts from healthcare providers. In order to ensure research rigour while conducting the study and in analysing all data, we employed the Consolidated Criteria for Reporting Qualitative research (COREQ).^27^

### Participant selection

The sample included 19 participants in Beirut, 6 women who accessed sexual health services, 8 women who did not, and 5 healthcare providers who deliver sexual health services from different disciplines: gynaecology/obstetrics, infectious disease, and urology. While we would have liked to interview a larger sample, accessing participants was difficult given the sensitive nature of the study.

In order to recruit women as SHC seekers, we sent an invitation letter to a large listserv (100+ people) for women accessing a sexual healthcare centre in Beirut. This list included healthcare providers who worked for the centre. The letter included a full description of the study and information on confidentiality and recruitment. Women who wanted to participate contacted the research team. We, therefore, used this convenience sample (i.e. the women who responded). Snowball sampling (i.e. word of mouth) was also used to recruit women who had not accessed care. Specifically, we began by interviewing women who had accessed sexual health care services. At the end of each interview, we asked if they knew women in their social networks who had not accessed such care and might be willing to participate in the study. Thus, the second group of participants was approached through an invitation relayed by the referring participant. If they expressed interest, the research team contacted them directly by phone to schedule an interview. Healthcare providers were selected more deliberately based on who was currently providing care at the sexual health centre. These providers were invited through email for a scheduled interview.

### Setting

Semi-structured interviews were conducted in Arabic, either face-to-face in a café or a sexual health centre, or online via Zoom or Webex, depending on the participants’ preferences. Participants were given the autonomy to choose locations where they felt comfortable expressing their thoughts, as some preferred not to visit the healthcare centre or participate in an online interview. Interviews were therefore conducted in participant-selected locations, with attention to quiet cafés during non-peak hours and ensuring adequate physical spacing. Despite some interviews taking place in cafés, we found that participants spoke openly and candidly about their experiences with sexual health.

### Data collection

The individual interviews for this study were conducted by the first author. Formal data collection was obtained between December 2022 and March 2023. Prior to the interviews, participants were briefed on the study’s purpose, the voluntary nature of their involvement, relevant ethical considerations, confidentiality, and data management. Oral verbal consent was obtained from all participants.

Women, whether service users or not, were initially asked about their perceptions of sexual health and sexual healthcare, factors encouraging or hindering access to SHC, and expectations from such services. Healthcare providers (HCPs) were asked about services provided in the centre, recurrent conditions seen, concerns of women seeking care, descriptions of service access, facilitators or challenges to seeking care, and recommendations to improve women's utilization of services. Interviews took between 30 and 45 minutes to complete; participants were not reimbursed for their time. (See the supplementary files for interview guides.)

Despite our relatively small pool of interviewees, we did reach some degree of data saturation in that codes were robust and repeated across the 19 interviews. Yet our study is by no means exhaustive. We see this as a baseline exploratory study upon which to build future work. We return to these points in the limitations section.

### Data analysis

All interviews were audio recorded and transcribed verbatim. Our data analysis process followed the approach outlined by Braun and Clarke^28^ for thematic analysis, in which the transcriptions were manually coded and analysed by two research members to identify relationships among recurring codes. This analysis was an iterative process, with codes being continuously reviewed and refined as data collection progressed. All codes emerged inductively from the interviews. Codes were then compared within and across interviews and were grouped into categories that were subsequently organised into subthemes and three overarching major themes. Our thematic analysis was done manually in Microsoft Word and Excel. No qualitative analysis programmes were used. To ensure the rigour of this study, emergent themes were discussed and refined in dialogue with all authors.

Our research team comprises three authors from the Middle East and North Africa region and one from the United States (US), including three women and one man, all of whom are married. Collectively, we bring disciplinary expertise spanning medicine and public health with a focus on sexual health. We recognise that our cultural backgrounds, gender identities, and marital status shape how we approach questions of women’s sexual health in Lebanon, influencing the topics we emphasise, the questions we ask, and the ways we interpret participants’ narratives. The authors from the region contributed contextual and cultural knowledge, which informed culturally sensitive recruitment strategies, phrasing of questions, and interpretation of nuanced experiences, while the US-based author brings comparative global perspectives, highlighting patterns and themes that resonate across contexts. Through ongoing dialogue, we critically reflected on how our positionalities could shape our framing, analysis, and interpretation, and we deliberately cross-checked interpretations to avoid imposing assumptions, ensuring that the voices and lived experiences of women in Lebanon guided the analysis throughout the research process.

### Ethical considerations

Prior to the start of the study’s data collection, ethical approval was obtained from the Institutional Review Board (IRB) of the American University of Beirut (AUB) (SBS-2022-0285) on 17/11/2022. Potential risks associated with participation in the study included the possibility of recalling distressing situations related to sexual healthcare. Participants provided voluntary consent and were orally informed about the purpose of the study, their right to participate or withdraw at any time, and the confidentiality of their information. Participants were identified using numerical coding according to the order of interviews, thus no personal names were added to the manuscripts. All information was securely stored on a password-protected computer.

## Results

This study explores perceptions of women around seeking SHC and their requirements from such services. We also examine healthcare providers’ perspectives to contextualise and better understand the service delivery factors that influence women’s experiences. In terms of demographics, the sample included 14 women living in Beirut aged between 20 and 50 years. Six women who accessed sexual health services were recruited from the first phase through the sexual healthcare centre listserv, and eight women who did not access sexual health services were recruited through snowballing. Ten women were married; three had children. All held a university degree with various occupational backgrounds. The sample also included five HCPs, four women and one man, who deliver sexual health services from different disciplines: three gynaecologists/ obstetricians, one infectious disease specialist, and one urologist. Socio-demographic characteristics of the women, including age, marriage, having children, education, work experience, and whether seeking SHC, are presented in Table 1. Work experience of healthcare providers, including specialty and years of experience, is presented in Table 2.

**Table 1.** Socio-demographic characteristics and sexual healthcare-seeking behavior of women in Beirut, Lebanon, 2022–2023

Participants	Age	Marriage	Children	Education level	Work experience	Seek SHC
P 1	26	Yes	No	University	Math teacher	No
P 2	27	Yes	No	University	Business discipline	No
P 3	28	Yes	Yes	University	Pharmacy	No
P 4	29	yes	No	University	Architect	No
P 5	28	yes	No	University	Medical Lab	No
P 6	29	yes	No	University	Business	Yes
P 7	28	no	No	University	Speech Therapist	No
P 8	36	yes	No	University	Business Research	Yes
P 9	35	yes	Yes	University	Sales Manager	Yes
P 10	48	yes	Yes	University	CEO Assistant	Yes
P 11	27	yes	No	University	Data Analysis	Yes
P 12	34	no	No	University	Social Enterprise	Yes
P 13	39	no	No	University	Research	No
P 14	24	no	No	University	Marketing	No

**Table 2.** Characteristics of HCPs, including gender, specialty, and years of experience in Beirut, Lebanon, 2022–2023

Participants	Gender	Specialty	Years of Experience
HCP 1	Female	Gynaecology; Obstetric; Sexology; Cosmetic Gynaecology	18
HCP 2	Male	Urology Consultant; Sexology	25
HCP 3	Female	Infectious disease physician; HIV expert; Director of the antimicrobial stewardship program	15
HCP 4	Female	Laparoscopic surgeries; Obstetrician & Gynaecologist; Cosmetic Gynaecology	22
HCP 5	Female	Clinical Sexology; Sexual Medicine	16

Three main themes were derived from the analysis. *Theme 1: Barriers to care* encompassed four subthemes: cultural and societal attitudes toward sexual health; fear of judgement and stigma; communication barriers with healthcare providers; and limited available services. *Theme 2: Facilitators to care* compromised five subthemes: general awareness and understanding of SHC services; initiation of sexual activity; enabling environment; having a medical condition as a trigger; and positive physician–patient communication. *Theme 3: Recommendations* included three subthemes: improved service availability and accessibility; enhanced marketing and awareness campaigns; and provider training in sensitive, non-judgmental care (Table 3).

**Table 3.** Emergent themes and sub-themes from interviews on perceptions of women on seeking SHC in Beirut, Lebanon-2022–2023

Themes	Sub-themes
1. Barriers to care	Cultural and societal attitudes toward SHC Fear of judgement and examination Communication barriers with healthcare providers Limited availability & affordable SHC services
2. Facilitators to care	General awareness and understanding of SHC services Initiation of sexual activity Enabling environment Having a medical condition as a trigger Positive physician–patient communication
3. Recommendations	Improved service availability and accessibility Enhanced marketing and awareness Provider training in sensitive, non-judgmental care

### Theme 1: barriers to care

Participants described various barriers that hindered or affected their access to SHC. The theme reflects diverse personal, deep-rooted cultural factors, and systemic challenges that created a climate of avoidance or discomfort related to service provision.

#### Cultural and societal attitudes toward SHC

All participants emphasised that prevailing cultural norms and societal attitudes precluded women’s ability to openly express their perceptions regarding sexual health. In a context where discussions around sexuality are considered taboo, as elaborated by all, sexual health is often practiced in silence, which discourages women from openly discussing how they access sexual healthcare. Women reported that their communities often perceive sexual healthcare as unnecessary or a low priority, particularly amid the multiple crises affecting the Lebanese population. These attitudes contribute to misinformation, limited knowledge, and hesitancy, delaying women’s access to sexual health services when they are needed.

*“In our community, it’s considered shameful to talk about sexual issues especially before marriage. Considering that it’s a sensitive topic, there’s a lot of embarrassment that discloses opening a dialogue on sexual health.”* (P1)

Given the sensitivity of this topic in the Lebanese context, women consistently spoke of barriers to care, including the “hidden”, “taboo”, and “shameful” nature of SHC.

*“People generally perceive sexual communication as private and hidden. While some are getting open to the topic, other people still conserve their traditions and values which hinder revealing intimate information and seeking care.”* (P8)

Several women described feeling that their sexual activity was supposed to be unflawed, easy, or perfect (i.e. not dysfunctional), making it shameful to seek care.

*“I don’t know why they fear from this, maybe because they think that they are sexually perfect and not ill as if it is a shame if someone has a problem, or I think that what really hinders them from seeking care is the societies’ judgement, I am sure if it wasn’t for the society, people would have gone and seek assistance or information.”* (P3)

Most healthcare providers confirmed that cultural and societal norms hindered women’s ability to seek SHC due to stigma, shame, and discomfort. This resulted in silence, a lack of knowledge and/or awareness, or being subjected to “misinformation.”

*“One of the challenges is talking about your sexuality is difficult because it’s intimate, which contributes to lack of information or misinformation.”* (HCP5)

*“I don’t know if they’re educated properly & uniformly in all schools about condoms, vaccines, screening, testing, happy life, saying no if they want to.”* (HCP3)

Care-seeking was also influenced by parents, families, and schools. All participants highlighted the discomfort that mothers feel in communicating with their daughters and a fear that discussions might increase their daughters’ awareness of sexual issues. Providers acknowledged that parents feel awkward having these discussions with their children.

#### Fear of judgement and examination

Women commonly discussed fears that barred them from accessing SHC. Fear of judgment was a central barrier. This included fears that a physician might judge them for being sexually active, especially if they were unmarried, or for a medical condition. Furthermore, women were embarrassed to show their private body parts and undergo a diagnosis. Those who had never been to an examination were afraid of the diagnostic procedures themselves.

*“There is a lot of judgement, and it is something intimate & private, or let’s say that if the doctor wants to undergo an examination and I don’t feel comfortable with the test, I will be afraid and embarrassed.”* (P1)

Two women who went for a check-up with a gynaecologist before marriage felt judged by the physician for choosing to take contraception. Women who access care feared being socially judged if seen at the clinic, so they preferred appointments to be spaced apart to avoid seeing other women in the waiting room.

Although the majority of women who sought SHC mentioned fear of examination and judgment before the visit, once they sought care, they became more confident and encouraged others to do so*.*

*“Service over-met my expectations. I was afraid at first because I haven’t done such a test before and haven’t seen a physician concerning this subject. I had a case, and I was afraid what he would tell me, but it was fine and smooth, he gave me needed information and made me feel comfortable, it was easy.”* (P11)

#### Communication barriers with healthcare providers

Physician–patient communication was a repeated concern for all women, whether they sought SHC or not. They emphasised that a negative or judgmental experience could create a challenge for follow-up visits or future concerns. One woman felt offended by her OBGYN for worries before an examination when her provider said: *“As if you are the first one to do that!!”* (P1)

Healthcare providers emphasised the limited capacity of some urologists, dermatologists, or sexual health specialists, such as even gynaecologists, to provide the appropriate counselling from a compassionate, sensitive place. A main complaint was the lack of training on delivering clear, professional, and educational counselling.

*“Provider-patient communication is really important, but the premise is the general healthcare providers or urologists or even dermatologists which I don’t think they know how to approach patients and to bridge that gap of delivering an information directly about sex matters.”* (HCP 5)

*“Because it’s an intimate topic so it is the same as going to a psychologist or psychiatrist, when you are talking about your feelings, about yourself, if you are not comfortable, you won’t get the treatment or result you want.”* (P14)

One woman who was a cancer survivor explained she would have preferred to be given appropriate counselling from her oncologist regarding the treatment consequences on her sex life, or to have been referred to a therapist.

*“When knowing about cancer, even family were in sorrow and I couldn’t think about sexual health, but you know what psychological issues raise after mastectomy like body acceptability, and after chemotherapy I had decreased libido which affected my sexual life. My oncologist and surgeon didn’t counsel about it, and I had to accept my body by myself.”* (P10)

#### Limited available and affordable SHC services

Healthcare providers emphasised that one of the reasons for low access to services is a lack of awareness or lack of availability of services, as well as a conflation between SHC and other gynaecological services.

*“I did not know about the availability of sexual health services in Lebanon, I knew about infectious specialist and gynecologist but not Sexual health specialist.”* (P5)

*“If people wanted to search for sexual health centers, they won’t easily find because it is not marketed for enough and not advertised.”* (HCP 2)

Cost of treatment was another addressed reason for the low access to services.

*“Maybe another reason is the financial, people feel it is not worth to spend money on it unlike having a physical illness as hypertension, diabetes, or stroke.”* (P3)

Among the six women who sought care and the eight who did not, we observed differences in their experiences. Women who accessed care described barriers related to service quality and providers’ attitudes that hindered effective physician–patient communication. In contrast, women who had not sought care emphasised anticipatory concerns, including fear of judgment, stigma, lack of confidentiality, cultural attitudes, and uncertainty about service availability.

Participants in both groups had similar socioeconomic status and educational backgrounds. However, even with our small sample, patterns emerged related to marital status. Unmarried women expressed particular concern about stigma and social repercussions of being sexually active, especially regarding confidentiality and disclosure, whereas married women focused more on challenges related to service quality and provider communication. We caution readers against drawing definitive conclusions from these differences, given the limited sample.

### Theme 2: facilitators of care

Our second theme highlights the factors that encourage women to access sexual health services as described by participants. Women who accessed care explained that general awareness and understanding of SHC services, as well as initiation of sexual activity, increased their likelihood to seek care. All women emphasised that a motivating environment, whether through supportive family, peers, or social media, facilitates talking about sexual health concerns. Others indicated that experiencing a medical condition serves as a trigger to seek professional care. Most importantly, positive physician–patient communication could be a major facilitator for ongoing supportive care.

#### General awareness and understanding of SHC services

The importance of sexual health literacy – specifically having foundational background knowledge in sexual health – was a powerful facilitator for care-seeking. The women we interviewed who had sought sexual health services spoke of this type of healthcare as integral to their overall health. In other words, women who had a baseline knowledge and were informed about the importance of SHC were more likely to use these services.

As described by one of the participants:

*“Honestly, I don’t know about others, but each woman should care about this topic, it’s particularly important, because if I am mentally or physically not comfortable, this [sexual health] will affect my health.”* (P7)

Above, Participant 7 describes the importance of caring about this topic. She explains how discomfort with sexual health affects overall health. In interviews, healthcare providers confirmed that SHC-seeking was more likely to happen when it was understood as important for women’s physical, mental, and psychological health.

Notably, for some, background knowledge on sexual health services was aligned with religion. Religion encourages/condones sexual activity when it’s bound under marriage.

*“Logically, being open-minded could facilitate talking about SHC because it is a science, if we need to talk in terms of religion, it is permissible to talk about it to a professional because this practice is a blessing from God.”* (P10)

Study participants also talked more generally about having education and knowledge of available services as important for seeking care. Two HCPs explained that SHC is starting to increase in part because newer generations are more knowledgeable and aware of sexual concerns. This finding aligns with women’s perceptions.

*“Access to service is getting better with sexual education and media awareness.”* (HCP 1)

*“One way to destigmatize the topic is to talk about it in social of media where people can become aware about sexual health.”* (HCP 5)

Participants noted that each sexually active woman has her own experience – and also that providers had the potential to be a trusted source for information. Thus, nearly all the women we interviewed preferred communicating with a physician to get reliable information rather than asking others from their family, community, or the internet.

*“My family is very conservative, I can’t communicate this information with them, even if my friends are open-minded if they aren’t the right people, I can’t also take consultation from them because every woman has her own experience, and I need someone professional and somehow being objective.”* (P2)

#### Initiation of sexual activity

Initiation of sexual activity was also named as a reason to seek sexual health services, serving more as a moment of recognition than as a factor that directly facilitated care-seeking.

Some participants noted that seeking care is traditionally associated with marriage and the onset of sexual activity, where they emphasised the importance of proactive health practices, like HPV vaccination and counselling on reproductive health and safe practices.

*“Initiation of sexual activity is an important factor to the increase of access to care because women realize they need counseling on vaccines, sexually transmitted infections, or contraception use.”* (HCP 2)

#### Enabling environment

All women, whether care seekers or not, described that having a motivating environment is a main driver for seeking SHC. Encouragement from parents, friends, spouse, or trusted networks created a supportive environment where women had confidence to make health decisions.

*“Mmm mainly people who want to start having sexual relationships, and if the surrounding environment encourages them for this; for me, if I am in a motivating family, I would go do this orientation before taking any step … so I should already have a background on the topic in order to seek care.”* (P2)

*“When I heard from the surrounding [environment] about someone who seek help, I felt encouraged to go.”* (P11)

#### Having a medical condition as a trigger

Most often a medical condition drove care-seeking. Being symptomatic or having unsatisfactory sexual activities made women more likely to seek care.

*“I would consult a specialist if I had an unplanned pregnancy or STI but as long as I am not having a serious condition I won’t.”* (P12)

Symptoms created a sense of urgency that overrode cultural and societal attitudes related to stigma or embarrassment. In such instances, care-seeking became a necessity. As one woman explained, waiting to seek care could exacerbate the medical condition or normalise it. Below, this quote highlights the role of health providers in addressing an issue like sexual pain, rather than minimising or normalising it.

*“I had everyday pain and thought it was normal because I was used to it. The doctor elaborated during the examination that being in pain should not be normal. Since then, I knew that when having symptoms, I should see a physician.”* (P5)

#### Positive physician–patient communication

Both women and healthcare providers stressed the need for respectful communication with healthcare providers/professionals to facilitate care-seeking. Women expressed a desire for specialists to address their mental and psychological concerns before focusing on physiological conditions or conducting medical exams, highlighting the importance of brief counselling to help them feel comfortable and at ease. They desired respectful, professional, and non-judgmental interactions. As portrayed by one woman, a physician’s counselling about a vaccine gave her confidence to protect her health.

*“My doctor initiated the discussion indirectly, when I went, he explained about the vaccine and cancer before I administer it, and I remember he clarified on everything related to women’s reproductive system and how sexual health is related to it. He gave back personal motive to be strong confident and not be embarrassed from my actions. I felt comfortable to seek care anytime I needed.”* (P8)

*“To be honest, despite being strong, self-confident, and reconciled with yourself you will be shy at first to be talking about a sensitive topic with a man; this is the way we got raised, but I was so happy & comfortable with the counseling, I mean wow it was super helpful, and I would definitely encourage others to go … he listened to all my concerns at first and provided the exact information I needed, he didn’t make me feel worried or anxious about next step … I trust him a lot and he was the one who helped build that trust.”* (P9)

As noted above, participants’ accounts suggested there were sometimes specific moments in women’s lives when they might seek sexual care. The importance of having a positive interaction with a healthcare provider around sexual health is heightened in these instances because the women do not seek care if there is no medical condition. When a sexual health condition was treated in a supportive healthcare environment, this could pave the way for further medical support and ongoing care-seeking.

### Theme 3: recommendations

All participants expressed the need to normalise conversations about sexuality, while integrating sexual health services within the broader health system to make these services accessible, affordable, socially acceptable, and free from stigma and judgment.

#### Improved service availability and accessibility

As noted by some women and healthcare providers, more funding, sliding-scale costs or payment plans might help lessen the burden of paying for sexual health. Participants noted that more efforts should be made at the primary healthcare level to make these services accessible.

*“I've been hearing about … it's sponsored by an NGO, where they are trying to alleviate and decrease the burden and to make services more accessible for girls or women, but I think we should do more on that level as health centers.”* (HCP 4)

#### Enhanced marketing and awareness

Participants suggested that the dissemination of targeted messages, such as *“women who are sexually active and experience the following symptoms should seek care at … ”* would significantly enhance women's awareness about various conditions and available services. Healthcare providers recommended the use of social media, TV, radio, and community-based outreach to help normalise these messages across diverse age groups and social classes. More formal marketing and advertising might also normalise these services in women’s support groups at the community level, where women can share experiences, ask questions, and be referred to services. Also, integration of SHC into broader health campaigns (e.g. maternal health, cancer screening, or chronic disease prevention). As noted by healthcare providers, this more empowered, proactive approach to public messaging can help address the knowledge gap and motivate women to take charge of their sexual health.

*“We can include SHC into broader campaigns, where we should have a list of referral centers clearly indicated with their costs. This will help inform people that services exist and improve utilization.”* (HCP 3)

#### Provider training in sensitive, non-judgmental care

Overall, women and HCPs stressed the need for respectful communication with healthcare providers/professionals and the need for more training for providers. Participants wanted women-friendly services that included discussions of sexuality in normalised, destigmatised, and non-judgmental ways. One woman suggested a national recommendation for annual check-ups for women at a certain age. Women believed that providers should first address their concerns before doing exams and lab tests. Participants emphasised the importance of continuous training to ensure that services promote gender equality and empower women to make decisions about their health.

*“If you want to improve the service, you need to improve the way physicians deal with patients, I think Drs need training about that. I see that the Dr’s attitude has a significant role in affecting access to healthcare, I don’t care about the clinic or its setting, I care about the how much the physician will make me comfortable and clearly explains in detail.”* (P5)

## Discussion

This study explores the perceptions and experiences of women living in Beirut regarding access to sexual healthcare and their expectations and needs related to these services. We also examine HCPs' perspectives to contextualise and better understand the service delivery factors that influence women’s experiences. While studies in the Arab world that assess women's perceptions and experiences in sexual healthcare are scarce, existing research suggests that women are generally dissatisfied with the available health services^9^ Our findings extend prior scholarship by providing a more nuanced analysis of barriers, facilitators, and recommendations for advancing sexual health, underscoring the potential benefits of positive experiences, systemic reform, and provider training.

The study revealed that cultural norms, fear of judgment, poor communication with providers, and lack of awareness remain central barriers to seeking sexual healthcare in Beirut. These findings are consistent with research across the Arab region that has highlighted how taboos surrounding sexuality, coupled with stigma and fear of judgment, discourage women from seeking necessary care and place them at higher risk of unsafe abortions and sexually transmitted infections.^29–31^ Similar barriers have also been documented in national surveys conducted in Tunisia, where dissatisfaction with services stemmed from poor client–provider relationships, lack of active listening, and unaddressed concerns.^32^ The present study contributes novel insight by showing that, despite such barriers, participants want to talk about their sexual and reproductive health with providers, they desire accessible care and consider positive experiences with providers to have a lasting impact. This recognition signals a degree of readiness among women in Beirut to engage with services if they are made more accessible and acceptable. In line with previous studies conducted in Lebanon that revealed a reluctance among women to discuss their difficulties with healthcare providers,^3^ we did find some hesitation among participants. However, our findings suggested that such reluctance could be overcome with supportive sexual health services. In fact, once they felt comfortable, Lebanese women were very interested in engaging in conversations about sexual health, and once women decided to address their sexual problems, they preferred consultations with healthcare providers rather than family or friends.

It is worth noting that provider biases, particularly regarding contraception use, emerged across interviews. Similar biases have been documented in the provision of sexual and reproductive health services for adolescents and refugees in various contexts, where access to contraception is restricted based on age and marital status.^33,34^ Our findings confirm existing qualitative work conducted with midwives and nurses in Lebanon, where some healthcare providers still had stereotypical and gendered attitudes that impacted their provision of care, while those most interested in sexual health were most willing to provide care.^23^ Both the providers and women whom we interviewed mentioned that general physicians and primary care doctors may not be adequately trained to address sexual issues (including sexual function and pleasure) with their patients.^35^ Similar concerns have been expressed in the literature, emphasising the need to enhance healthcare providers’ training to strengthen their skills in delivering sexual healthcare services.^23,36^ Our participants also discussed the limited availability of sexual health services throughout the country, which is consistent with a previous needs assessment conducted in Lebanon, which identified fewer than five sexual health services accessible to the population.^14^ These gaps may worsen given the current mounting crisis in the region.

Participants also identified several facilitators that enabled access to care. General awareness of sexual health, enabling environments, having a medical condition, and positive provider communication encouraged help-seeking, echoing findings from previous studies in the Arab region that emphasise the importance of health literacy and non-judgmental provider attitudes.^35,37^ Unique to this study, however, was the identification of medical conditions as a trigger for accessing sexual healthcare. For instance, one participant described how her breast cancer treatment significantly affected her sexual health and relationships, illustrating how chronic illness and its side effects can prompt the need for sensitive discussions around sexuality even from specialties other than sexual health. In line with past research,^35^ this participant's account points to the importance of sexual health for people’s lives, relationships, and well-being, even in the midst of other medical care such as cancer treatment. In her story, the changes in her body (mastectomy) and impacts on libido (from chemotherapy) impacted her sexual health. Such evidence remains underexplored in the literature on the Arab region. This finding highlights the importance of integrating sexual health counselling into broader medical care pathways, particularly for women undergoing treatments that impact sexual well-being.

As noted in the results, important variations emerged between women who had sought care and those who had not. Women who accessed care described barriers related to service quality and providers’ attitudes, while those who had not sought care emphasised anticipatory concerns, including cultural attitudes, fear of judgment, stigma, lack of confidentiality, and uncertainty about service availability. Unmarried women were particularly concerned about stigma and social repercussions of being sexually active, especially regarding confidentiality, whereas married women focused more on service quality and provider communication. These findings underscore that barriers to sexual healthcare operate at multiple levels, highlighting the need for interventions addressing both social and health-system factors.

Both women and healthcare providers in this study recommended strengthening sexual healthcare in Lebanon, through increasing the availability of services, raising awareness, and enhancing provider training. These recommendations mirror international calls for women-friendly, stigma-free sexual and reproductive healthcare services^3,37^ and align with national-level evidence from Tunisia, which revealed dissatisfaction with services due to inequitable treatment, inadequate follow-up, and poor responsiveness to women’s needs.^32^ Findings suggest that more sexual health literacy for patients and education/training for providers might be tremendously beneficial in this population as a means to promote sexual health and sexual rights. Perspectives from both women and providers in Beirut reveal a similar set of priorities, suggesting a need for system-level reforms whereby policymakers and stakeholders might strengthen sexual health services in ways that are community-driven and provider-supported.

Our findings suggest great potential for services supportive of sexual health and sexual rights in Lebanon precisely because this topic is sensitive in the country. In particular, healthcare providers in the study emphasised that access to sexual healthcare is currently low but has been gaining visibility.^6^ The study points to a promising moment in Lebanon for fostering lasting change in the normalisation of sexual health services.

### Limitations

This study has several limitations. First, the small sample size, restricted to women in Beirut and largely from similar higher socioeconomic backgrounds, limits the generalisability of the findings. While participants frequently highlighted financial constraints as a barrier to care – consistent with other research – our reliance on a relatively affluent sample may have led us to underestimate the extent of cost-related barriers among women from lower-income groups. Of note, our sample was entirely university-educated, which may have limited our ability to fully capture barriers related to health literacy, awareness, or understanding of sexual health services that women with lower educational levels might experience. Second, the use of a convenience sample may have introduced selection bias, as women who agreed to participate may have been more open to discussing sexual health than those who declined. This could mean our findings overestimate women’s willingness to engage with providers on these issues. Third, our findings may over- or underestimate the prevalence of certain views or experiences. To mitigate this, interviews were designed to probe for detail and consistency. Future quantitative studies could help validate some of these qualitative findings by using multiple measures or triangulating across methods. Despite these limitations, the study provides valuable in-depth insights into barriers, facilitators, and opportunities for improving women’s access to sexual health care in Lebanon. Our study also increased awareness for the women who had not accessed care. These women became aware of the availability of SHC care in Beirut after study participation.

### Recommendations

We propose the following recommendations based on our findings to address the challenges and gaps in SHC services in Lebanon:

#### Service provision level

- Integrated services should be implemented, where sensitive healthcare providers deliver care in a stigma-free and safe environment that guarantees privacy and confidentiality.
- Effective marketing of sexual health services is crucial, utilising media and accessible public spaces to promote education and awareness. Healthcare providers can play a key role in transmitting messages through social marketing campaigns. For example, incorporating messages such as: “If you are (age) and (sexually active), whether married or not, and experiencing (specific conditions), visit this programme or contact the following … ” and disseminating such messages would enhance women's knowledge about relevant conditions and available services.
- Training and capacity-building programmes should be developed for healthcare providers interested in sexual health. These programmes should focus on improving communication skills and adopting a patient-centred approach in delivering sexual health services. Training opportunities can be extended to both pre-graduate and in-service providers.
- Establishing clear referral pathways between gynaecologists, oncologists, urologists, and dermatologists is essential to ensure comprehensive care for patients.

#### At a national level

- Efforts should be made to normalise and publicise discussions about sexual health as an essential service that individuals should seek whenever they require healthcare. This can help reduce stigma and promote a proactive approach to sexual health.
- Advocacy initiatives should be undertaken to integrate comprehensive sexual and reproductive health services into Primary Healthcare Centres. This can be achieved through the development and implementation of supportive policies and guidelines.
- Coordination with the Ministry of Education and Higher Education to incorporate a comprehensive age-appropriate sexuality education programmes in schools.

#### At the community level

- Establish women’s support groups that can serve as a trusted source for peer learning, emotional support, and referral to services. These groups can be facilitated by community health workers and volunteers who normalise discussion about sexual health and increase awareness of seeking care.

## Conclusion

This study offers new insights into how women in Lebanon perceive and experience sexual health, highlighting both persistent barriers and opportunities for progress. Participants described how cultural norms, fear of judgment, communication challenges with providers, and lack of awareness continue to limit access to care, echoing barriers documented across the Arab region. At the same time, key facilitators were identified – including greater awareness of services, supportive environments, medical conditions prompting care-seeking, and positive physician–patient communication – demonstrating that engagement with sexual health services is possible when the context is supportive. Both women and providers emphasised practical recommendations for strengthening sexual healthcare in Lebanon, including expanded service availability, awareness-raising, and provider training in sensitive, non-judgmental care. Together, these findings suggest that, despite entrenched stigma and systemic constraints, women in Beirut are ready to engage in sexual health discussions if services are made more accessible, acceptable, and supportive.

Beyond informing research, the study itself may have contributed to community awareness, as some participants reported learning about the availability of specialised services during the interviews and intended to share this information with others. These findings have several implications: for practice, provider training is essential to normalise sexual health discussions in routine care; for policy, systemic efforts are needed to expand equitable and integrated services; and for research, larger, mixed-method studies can build on these insights to design and evaluate interventions.

Amid ongoing regional instability, challenges to equitable access, integration, and sustainability of women-friendly services may intensify. Yet this moment also presents a critical opportunity: by fostering an enabling environment that promotes education, awareness, and open dialogue, Lebanon can strengthen sexual health services, prevent STIs and unintended pregnancies, combat gender-based violence, and advance sexual rights for all. A respectful, inclusive approach to sexuality and care can dismantle stigma, expand access, and ultimately improve women’s overall well-being.

## Supplementary Material

Interview Guide
