# Supplementary material for: Perceptions of women on seeking sexual healthcare in Beirut: a qualitative study
Source: Sex Reprod Health Matters. 2026 Mar 4;33(1):2639175. doi: 10.1080/26410397.2026.2639175 (PMC13063322; doi:10.1080/26410397.2026.2639175)
Supplement: Interview Guide [file ZRHM_A_2639175_SM2602.docx]

**Interview guide on Perceptions of Women on Seeking Sexual Healthcare in Beirut: A Qualitative Study**

**:**

**Before the start of the interview, mention to participants:**

-All information discussed should be kept confidential and should not be disclosed to anyone.

-Refraining from stating any sensitive information or any information that may identify specific incidents/individuals.

-Refrain from mentioning the name of your or any organization/institution.

**With women:**

**Ice breaking: tell me a little bit about yourself and what services do you go to how often?**

1. Can we talk about sexual health, what do people think about it?
2. Where do people go if they need help for sexual health matters?
3. What factors might encourage them to seek care? What hinders them from doing so?

-Probe on environment, context, beliefs, norms

1. What does sexual health mean to you?
2. If you had to seek care for sexual health, what would you expect from these services?

- what do you want from these services to ensure your confidentiality, and privacy as literature has shown that these factors hinder women from access to care- (this question is meant to understand factors identified by literature behind women not seeking care and not triggering a feeling of embarrassment or discomfort)

1. What do you like to see in such services that will help people resolve sexual issues?

**With healthcare providers:**

**Ice breaking: tell me a little bit about your career, for how many years have you been working in the center?**

1. What services are provided at the center?
2. What are the issues that you see most often that relate to sexual health?
3. From your experience what are the emerged concerns of women about seeking care?
4. How do you describe the access to these services?

-who comes, client’s numbers?

1. What are some facilitators and challenges that people are dealing with?
2. What do you think should be done to improve uptake or people utilizing these sexual health services?
